# Effect of Short Time of SARS-CoV-2 Infection in Caco-2 Cells

**DOI:** 10.3390/v14040704

**Published:** 2022-03-29

**Authors:** Luisa Zupin, Francesco Fontana, Libera Clemente, Maurizio Ruscio, Giuseppe Ricci, Sergio Crovella

**Affiliations:** 1 Institute for Maternal and Child Health IRCCS Burlo Garofolo, 34137 Trieste, Italy; giuseppe.ricci@burlo.trieste.it; 2 Division of Laboratory Medicine, University Hospital Giuliano Isontina, 34149 Trieste, Italy; francesco.fontana@asugi.sanita.fvg.it (F.F.); libera.clemente@asugi.sanita.fvg.it (L.C.); maurizio.ruscio@asugi.salita.fvg.it (M.R.); 3 Department of Medicine, Surgery and Health Sciences, University of Trieste, 34129 Trieste, Italy; 4 Biological Science Program, Department of Biological and Environmental Science, College of Arts and Sciences, University of Qatar, Doha 2713, Qatar; sgrovella@qu.edu.qa

**Keywords:** SARS-CoV-2, Caco-2 cell line, short time of infection, viral absorption

## Abstract

Coronavirus disease 19 (COVID-19) clinical manifestations include the involvement of the gastrointestinal tract, affecting around 10% of severe acute respiratory syndrome coronavirus 2 (SARS-CoV-2)-infected children. In the present work, the consequence of a short time of viral absorption (5, 15, 30 and 60 min) was tested on the Caco-2 intestinal epithelial cell line. Our findings show that Caco-2 cells are highly permissive to SARS-CoV-2 infection, even after 5 min of viral inoculation at a multiplicity of infection of 0.1. No cytopathic effect was evident during the subsequent 7 days of monitoring; nevertheless, the immunofluorescence staining for the viral nucleocapsid confirmed the presence of intracellular SARS-CoV-2. Our findings highlight the very short time during which SARS-CoV-2 is able to infect these cells in vitro.

## 1. Introduction

Severe acute respiratory syndrome coronavirus 2 (SARS-CoV-2) primarily affects the respiratory system; however, there is evidence that the gastrointestinal (GI) tract is also involved as diarrhea, nausea/vomiting, anorexia, and abdominal pain are common symptoms [1]. Intriguingly, it has been reported that children more often experienced GI features, since 25% of the children with acute SARS-CoV-2 infection developed diarrhea, nausea, vomiting, or abdominal pain [2]. Moreover, the occurrence of GI symptoms at the disease onset are unfavorably associated with a severe COVID-19 form; notably, in 90% of the multisystem inflammatory syndrome (MIS-C) cases, the digestive tract is involved [3]. SARS-CoV-2 presents a specific tropism for cells expressing the angiotensin converting enzyme 2 (ACE2) and the transmembrane serine protease 2 (TMPRSS2) [4,5], which are both present in the organs of the GI system [6].

This specificity is also reflected by the susceptibility towards SARS-CoV-2 infection restricted to few cell lines in vitro. Notably, the availability of permissive cell lines is mandatory for the characterization of the viruses, for the study of viral replication machinery, for the testing of potential antiviral drugs, and for vaccine development.

The epithelial Vero cell line from the kidney of *Cercopithecus aethiops* is considered the gold standard for virus propagation and studies on infectivity [7]; nevertheless, it is derived from a non-human primate and so potential differences with respect to the human-derived cell line may be expected [8]. Among the human cell lines, the Caco-2 colon epithelial cell line supports viral replication, although a cytopathic effect has not been detected [9].

Since it has been reported that Vero E6 cells were very susceptible to SARS-CoV-2 infection, even with a very low time of viral absorption (some minutes) [8], in the present work, we aimed at replicating and validating in a human cell model the findings already reported in the literature for Vero E6 cells. So, Caco-2 cells were subjected to a short time of viral inoculation at different viral concentrations; then, SARS-CoV-2 replication was monitored for 7 days post infection, assessing viral RNA in the supernatants and intracellularly. The presence of the virus inside the cells was confirmed by immunofluorescent analysis for the nucleocapsid protein.

## 2. Materials and Methods

### 2.1. Cell Line and SARS-CoV-2 Detection

Caco-2 epithelial cell line derived from colorectal adenocarcinoma (ATCC HTB-37) was maintained in Minimum Essential Medium (MEM) + 20% fetal bovine serum, 2 mM glutamine, and 100 U/mL penicillin/streptomycin (Euroclone, Pero, Italy); meanwhile, for the assays the cells were cultured in infection medium (MEM + 2% fetal bovine serum, 2 mM glutamine, and 100 U/mL penicillin/streptomycin).

All the experiments were conducted in 3 replicates.

The cells were seeded at a cellular density of 50,000 cells in 24 multiwell plates for the experiment of infection. The virus was isolated at the end of the first wave of the SARS-CoV-2 pandemic (summer 2020) [10].

The virus was tested for the major circulant variants by employing the SARS-CoV-2 VARIANTS REALTIME PCR KITS (detecting B.1.1.7, B.1.351, B.1.1.28.1 (P.1), and B.1.525 strains; Vircell Microbiologist, Granada, Spain), and the REALQUALITY SARS-CoV-2 AIM Variants (detecting AY.1/AY.2, B.1.617.2/B.1.617.1/B.1.617.3, and B.351/B.351.2/B.351.3 strains, AB Analitica, Padua, Italy) and for the 214 EPE insertion (characteristic for B.1.1.529 variant [11]) and was negative for the major variants circulating.

SARS-CoV-2 was diluted in the infection medium at a multiplicity of infection (MOI) of 0.1, 0.01, 0.001, and 0.0001, and then the viral absorption was carried out for 60′, 30′, 15′, or 5′ at 37 °C 5% CO_2_. After the different time points, the supernatants were discharged, the wells were washed in phosphate-buffered saline (PBS), and 1 mL of new infection medium was added to the cells.

On the 7th day, photos of each well were collected at 10× magnification to monitor the cytopathic effect with the EVOS XL Core Cell Imaging System (Thermo Fisher Scientific, Waltham, MA, USA). 

### 2.2. Viability Assay

The viability was determined by using crystal violet staining. At the end of the 7 days, the cells were washed in PBS and stained with crystal violet 10% in PBS for 30′, then the wells were washed in water and air dried. The staining was solubilized in SDS 1% and the absorbance read at 600 nm.

### 2.3. Immunofluorescence Staining

For the immunofluorescence assays, 15,000 Caco-2 cells were seeded in black 96 multiwell plates (Primo, Euroclone).

The same experimental procedure above described was followed.

At the end of the 7 days, the cells were washed in PBS and fixed in 4% paraformaldehyde diluted in PBS for 20 min.

The cells were permeabilized with PBS + triton-X100 0.2% (PBS-T) and then blocked with PBST + normal goat serum (NGS) 10%. The cells were incubated overnight with anti SARS/SARS-CoV-2 Coronavirus Nucleocapsid Antibody (MA1-7403, Invitrogen; Thermo Fisher Scientific, Waltham, MA, USA) (1:100 in PBST + 1.5% NGS), followed by 1 h incubation with anti-mouse Alexa Fluor 488 secondary antibody (A11029, Invitrogen; Thermo Fisher Scientific, dilution 1:500) and with Phalloidin for the staining of cytoskeleton F-actin (1:400, A12380, Invitrogen; Thermo Fisher Scientific). Finally, 30 μL of a mounting medium containing 40, 6-diamidino-2-phenylindole (DAPI) (Fluoroshield™ with DAPI, F6057, Merck KGaA, Darmstadt, Germany) was added to the wells and the image captured with the Cytation 5 Cell Imaging Multi-Mode Reader (Biotek, Winooski, VT, USA) at 10× and 60×.

### 2.4. Viral Load Quantification

After 0, 3, 5 and 7 days, 15 μL of supernatants was thermolyzed (98 °C for 3′ and 4 °C for 5′) in 45 μL of distilled water for RNA extraction. On the 7th day, the cells were also lysed in 100 μL of lysis buffer (10 mM Tris pH 7.4, 0.25% triton-X100, and 150 mM NaCl) for 10 min.

Then, the viral load was quantified by testing 3.5 μL of the supernatant lysates or 1 μL of the cellular lysates by real-time (RT) semiquantitative PCR, employing a previously quantified nCoV-CDC-Control Plasmid (Eurofins Genomics, Ebersberg, Germany) as standard. 

The Luna Universal Probe One-Step RT-qPCR Kit (New England Biolabs, Ipswich, MA, USA) and the CDC primers and probe (Eurofins) for the viral gene N (nucleocapsid, N1 set, 500 nM forward primer GAC CCC AAA ATC AGC GAA AT, 500 nM reverse primer TCT GGT TAC TGC CAG TTG AAT CTG, 125 nM probe FAM-ACC CCG CAT TAC GTT TGG TGG ACC-BHQ1) [12] were used on the 7500 Fast Real-Time PCR system (Thermo Fisher Scientific, Waltham, MA, USA, protocol: 50 °C for 10′, 95 °C for 1′, and then 40 cycles at 95° for 10″, 60° for 30″).

To confirm the viral productive replication, the ratio between subgenomic RNA for E gene and E gene was determined. The following primers and probe were employed for E gene: forward primer ACAGGTACGTTAATAGTTAATAGCGT (400 nM), reverse primer ATATTGCAGCAGTACGCACACA (400 nM), probe FAM-ACACTAGCCATCCTTACTGCGCTTCG-BBQ (200 nM) [13], while for the detection of subgenomic E gene RNA the forward primer was replaced by the subgenomic Lead SARS-CoV-2 forward primer CGATCTCTTGTAGATCTGTTCTC (400 nM) [14]. The Luna Universal Probe One-Step RT-qPCR Kit (New England Biolabs) and the 7500 Fast Real-Time PCR system (Thermo Fisher Scientific) were used with the same amplification protocol above described.

### 2.5. Statistical Analisys

The Kruskal–Wallis (KW) non-parametric test was employed to compare the different time points of viral replication using R statistical software [15]. Each experimental setting was performed in 3 replicates. 

## 3. Results

**Figure 1:**
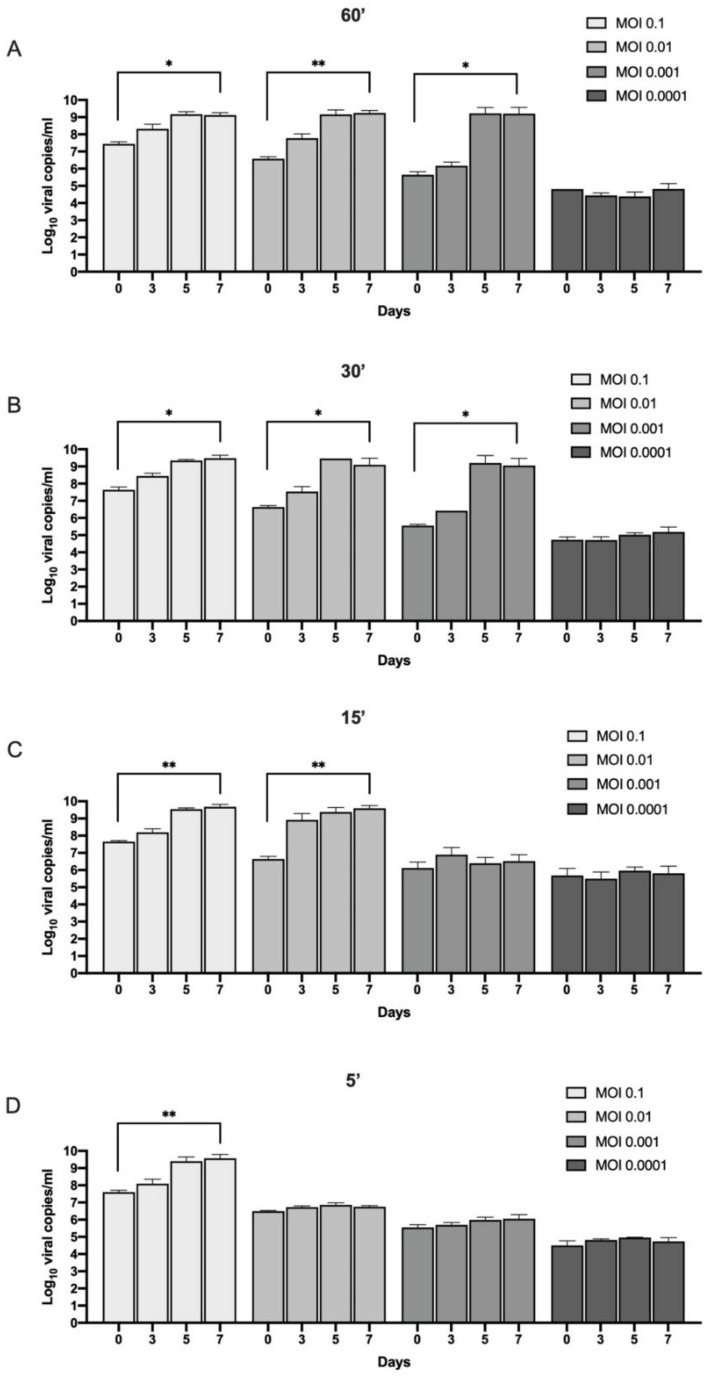
The RNA viral load (expressed as Log10 RNA viral copies/mL) at 0, 3, 5 and 7 day post infection at the different MOI (0.1, 0.01, 0.001, 0.0001) and time of absorption ((**A**) 60 min; (**B**) 30 min; (**C**) 15 min; (**D**) 5 min). Results from Kruskal–Wallis test are displayed. ** *p* < 0.01, * *p* < 0.05.

Viral replication was obtained by quantification of the supernatants for all the tested timings (60, 30, 15, and 5 min) with MOI 0.1. MOI 0.01 generated a productive infection with 60, 30, or 15 min of viral inoculation, while MOI 0.001 led to viral replication only with 30 or 60 min of viral adhesion (Figure 1A–D).

**Figure 2:**
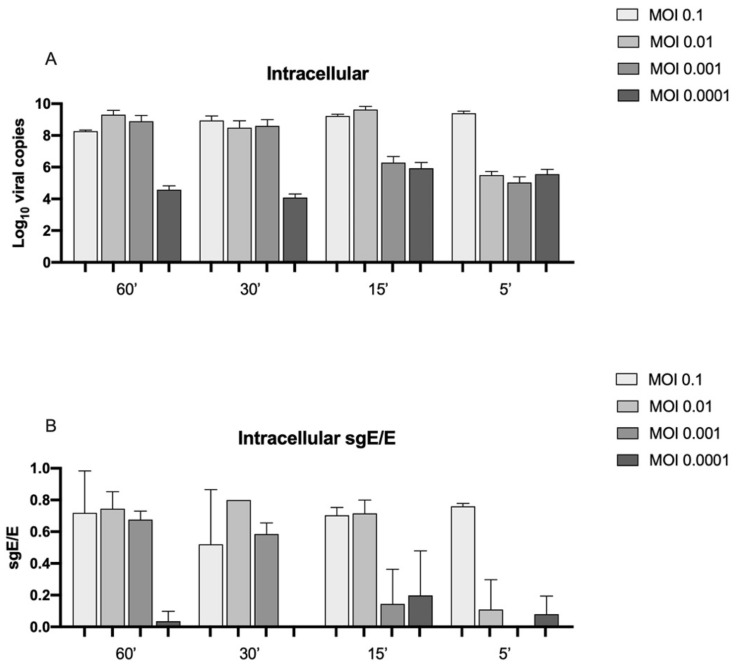
The intracellular RNA viral load. (**A**) Intracellular viral load for N gene (expressed as Log10 RNA viral copies) at 7 days post infection at the different MOI (0.1, 0.01, 0.001, and 0.0001) and time of absorption (60, 30, 15, and 5 min). (**B**) Intracellular ratio of subgenomic E RNA viral copies (Log_10_)/genomic E RNA viral copies (Log_10_).

The results were confirmed by the measurement of intracellular viral load at day 7 (Figure 2). Indeed, 10^8^–10^9^ RNA viral copies were determined for the experimental conditions where viral amplification was found; instead, for the experimental settings wherein SARS-CoV-2 did not generate a productive infection, the intracellular viral load was lower (10^4^–10^5^). To corroborate these data, the subgenomic RNA for E transcript was assessed. Subgenomic RNA could be considered as an indication for replicative virions; indeed, subgenomic RNA is transcribed only in actively infected cells and is still not packaged into virions [14]. The ratios of subgenomic E RNA on the genomic E RNA viral copies are reported in Figure 2B and corroborate the results of productive infection observed with the quantification of the supernatants.

Table 1 summarizes the results obtained. The average viral load in the supernatants is displayed, showing in light blue the experimental settings where successful viral replication was detected.

**Figure 3:**
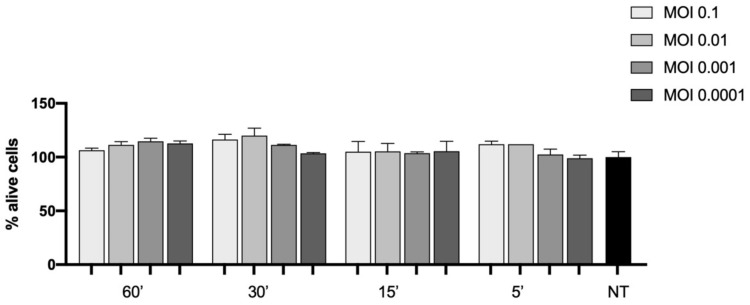
Viability of the cells infected with different virus concentrations (MOI 0.1, 0.01, 0.001, and 0.0001) and different timings of viral absorption (60, 30, 15, and 5 min) at 7 days post infection. The results are presented as percentage with respect to the cells not treated (indicated as NT in black).

Despite the susceptibility towards SARS-CoV-2 infection, no alteration in the viability was observed, even at 7 days post viral inoculation (Figure 3).

**Figure 4:**
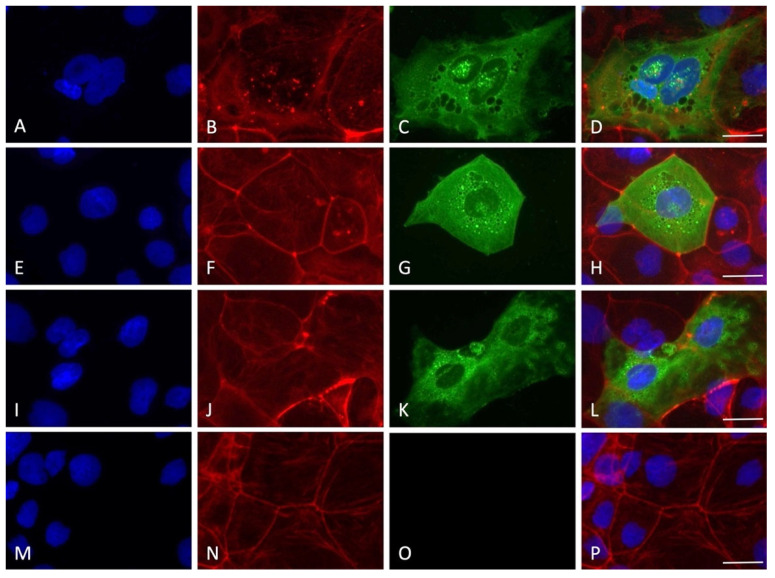
Immunofluorescence staining for nucleocapsid SARS-CoV-2 protein with the experimental protocol employing 60 min of viral absorption (60× magnification, scale bar 30 micron). The fluorescence for nucleus (blue), F-actin cytoskeleton (red), nucleocapsid protein of SARS-CoV-2 (green) and the merge of the three channels are displayed. (**A**–**D**) MOI 0.1; (**E**–**H**) MOI 0.01; (**I**–**L**) MOI 0.001; (**M**–**P**) MOI 0.0001.

**Figure 5:**
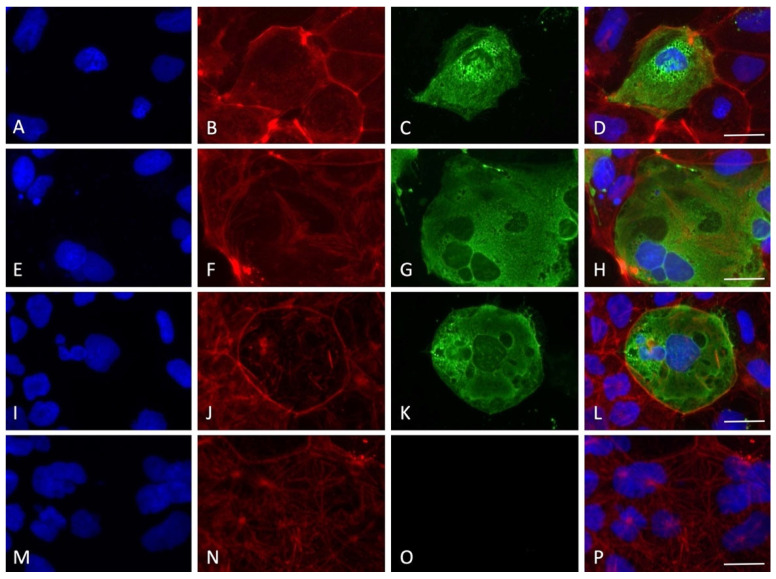
Immunofluorescence staining for nucleocapsid SARS-CoV-2 protein with the experimental protocol employing 30 min of viral absorption (60× magnification, scale bar 30 micron). The fluorescence for nucleus (blue), F-actin cytoskeleton (red), nucleocapsid protein of SARS-CoV-2 (green) and the merge of the three channels are displayed. (**A**–**D**) MOI 0.1; (**E**–**H**) MOI 0.01; (**I**–**L**) MOI 0.001; (**M**–**P**) MOI 0.0001.

**Figure 6:**
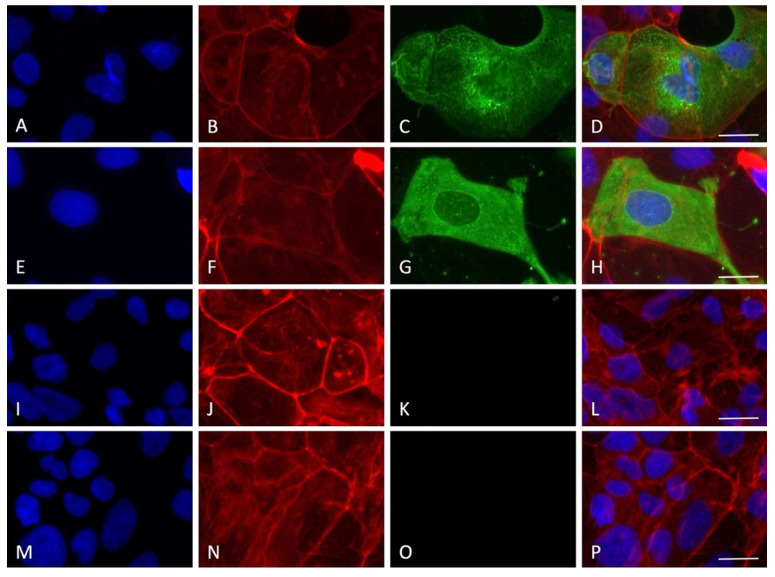
Immunofluorescence staining for nucleocapsid SARS-CoV-2 protein with the experimental protocol employing 15 min of viral absorption (60× magnification, scale bar 30 micron). The fluorescence for nucleus (blue), F-actin cytoskeleton (red), nucleocapsid protein of SARS-CoV-2 (green) and the merge of the three channels are displayed. (**A**–**D**) MOI 0.1; (**E**–**H**) MOI 0.01; (**I**–**L**) MOI 0.001; (**M**–**P**) MOI 0.0001.

**Figure 7:**
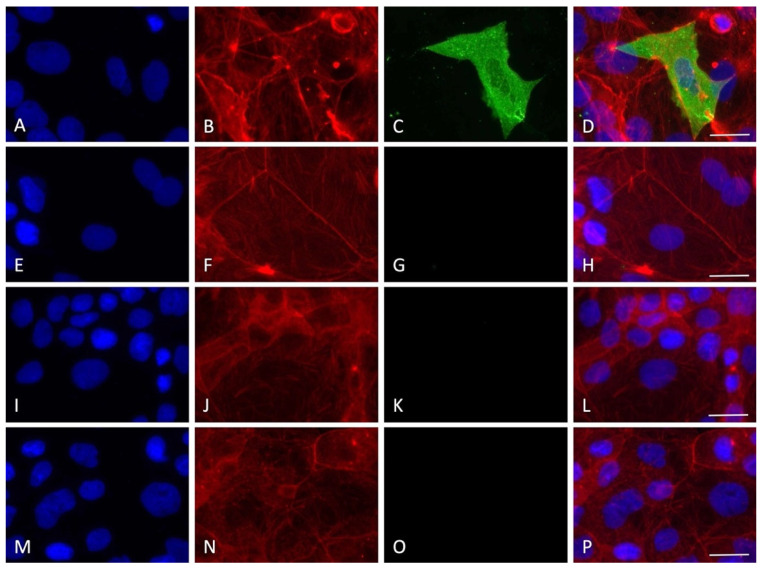
Immunofluorescence staining for nucleocapsid SARS-CoV-2 protein with the experimental protocol employing 5 min of viral absorption (60× magnification, scale bar 30 micron). The fluorescence for nucleus (blue), F-actin cytoskeleton (red), nucleocapsid protein of SARS-CoV-2 (green) and the merge of the three channels are displayed. (**A**–**D**) MOI 0.1; (**E**–**H**) MOI 0.01; (**I**–**L**) MOI 0.001; (**M**–**P**) MOI 0.0001.

**Figure 8:**
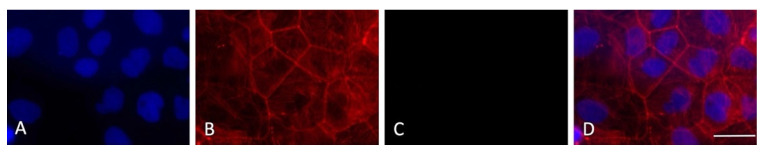
Immunofluorescence staining for nucleocapsid SARS-CoV-2 protein cells not treated (60× magnification, scale bar 30 micron). The fluorescence for nucleus (blue, (**A**)), F-actin cytoskeleton (red, (**B**)), nucleocapsid protein of SARS-CoV-2 (green, (**C**)) and the merge of the three channels are displayed (**D**).

By employing immunofluorescence staining for the nucleocapsid SARS-CoV-2 protein, the presence of the virus inside the cells was detected for all the timings of viral absorption positive at the RT-PCR quantification test. Interestingly, only some cells were positive to the staining, indicating cellular clusters of virus replication rather than a uniform infection in the monolayer, with an average of 10–15% of infected cells for all the experimental conditions tested (Figure 4, Figure 5, Figure 6, Figure 7 and Figure 8).

## 4. Discussion

The extrapulmonary symptoms of SARS-CoV-2 are multiple, e.g., diarrhea, vomiting, and abdominal pain, being common in pediatric subjects [2]. The tropism for the gastrointestinal tract reflects the specific SARS-CoV-2 virulence in vitro, preferentially restricted to few cell lines. Indeed, SARS-CoV-2 is able to enter and replicate in the Huh7 (hepatocellular carcinoma), 293T (embryonic kidney), Calu-3 (from lung adenocarcinoma), and the Caco-2 (from colorectal adenocarcinoma) human epithelial cell lines, Calu-3 and Caco-2 being the most permissive to infection [16]. This is in line with the main effects that SARS-CoV-2 triggers in the human host, such as the respiratory and the intestinal symptoms. Nevertheless, in other cell lines, such as A549 (alveolar epithelial cells from lung carcinoma), NCI-H292 (epithelial cells from lung mucoepidermoid pulmonary carcinoma), and RPMI 2650 (nasal epithelial cells), SARS-CoV-2 was not able to replicate [17]. All these cell lines are tumoral, so a different behavior with respect to primary cells or in vivo conditions can be reasonably expected, as, for example, in terms of the expression of the protein required for the virus entry and replication machinery [16]. Moreover, the tumoral cells, by their nature, are generally quite robust cells, so it could be plausible that they might also be less susceptible to infection.

However, our findings show that Caco-2 cells are permissive to SARS-CoV-2 infection even when only 5 min of viral absorption at a MOI of 0.1 was tested. An increment in SARS-CoV-2 RNA in the supernatants was observed during the 7 days of monitoring. The data were confirmed by the quantification of SARS-CoV-2 RNA intracellularly and corroborated by the analysis of subgenomic RNA for the E gene. Indeed, subgenomic RNAs are the intermediates for the translation of the viral proteins and are considered an index of active viral replication machinery inside the cells [18].

Interestingly, although the virus replicated in this cell line, no clear cytopathic effect was detected, a possible indication of the resistance of the Caco-2 cells that can sustain the viral machinery without apparent cellular damage. In order to prove the entry of the virus into the cells, immunofluorescence staining for the nucleocapsid protein was performed. A very specific presence of the virus in clusters of cells was observed; notably, SARS-CoV-2-positive cells presented a high level of nucleoprotein, diffused in all the cytoplasm with several spots of intense staining. Only a small percentage of the cells were found to be infected (10–15%), in line with previous results showing that 10% of Caco-2 cells were SARS-CoV-2 positive with a MOI of 0.1 at 24 h, also not showing a great increment in intracellular virus at 120 h post infection [19]. The lack of cytolysis could be due to the weak proinflammatory response roused by SARS-CoV-2. Indeed, it has been shown that the Interferon gamma-induced protein 10 (IP-10) was upregulated during in vitro infection, but not Tumor Necrosis Factor-alpha, RANTES (regulated on activation, normal T cell expressed and secreted), Interleukin 6, or Interleukin 8. Moreover, the type I and II interferon responses, which are the primary cellular reaction against the virus before the activation of adaptive immunity, were also not activated [19]. Therefore, the authors suggested that the virus is probably able to counteract the IFN pathways, leading to an attenuation of the host’s innate immune response.

Our observations were also in agreement with those reported by Chu et al., showing no cytopathic effect and also no change in cellular viability, though not with the study by Bojkova et al., that detected a cytopathic effect even at 24 h post virus inoculation [20].

The viral entry of SARS-CoV-2 is dependent on the interaction between the spike protein with the host ACE2 and on the cleavage of Spike protein by cellular proteases such as TMPRSS2 and furin [4,5]. Curiously, the human protein atlas reports for Caco-2 cells show no expression of ACE2, but a high level of TMPRSS2 and furin (normalized expression 14.1 and 12.9, respectively) [6]. However, the presence of ACE2 on the apical plasma membrane of Caco-2 has been detected in other studies, thus supporting the susceptibility of this cell line to SARS-CoV-2 [21].

Being aware that in vitro models are just a mimicking of real conditions, our findings place attention on the very short time in which SARS-CoV-2 is able to infect Caco-2.

## Figures and Tables

**Table 1:** The RNA viral load (expressed as average viral copies for ml of 3 experimental replicates) at 7 days post infection and at the different MOI (multiplicity of infection 0.1, 0.01, 0.001, and 0.0001) and timing tested (5, 15, 30, and 60 min). In light blue are displayed the experimental settings where an established infection was observed.

	TIME	5′	15′	30′	60′
MOI					
0.1		3.7 × 10^9^	4.7 × 10^9^	2.0 × 10^9^	1.3 × 10^9^
0.01		5.7 × 10^6^	3.9 × 10^9^	1.2 × 10^9^	1.8 × 10^9^
0.001		1.1 × 10^6^	3.3 × 10^6^	1.1 × 10^9^	1.6 × 10^9^
0.0001		5.5 × 10^4^	6.5 × 10^5^	1.5 × 10^5^	6.5 × 10^4^

## Data Availability

All the data used to support the findings in this study are included in the article.
